# Downregulation of LNMAS orchestrates partial EMT and immune escape from macrophage phagocytosis to promote lymph node metastasis of cervical cancer

**DOI:** 10.1038/s41388-022-02202-3

**Published:** 2022-02-12

**Authors:** Yuandong Liao, Jiaming Huang, Pan Liu, Chunyu Zhang, Junxiu Liu, Meng Xia, Chunliang Shang, Shiyin Ooi, Yili Chen, Shuhang Qin, Qiqiao Du, Tianyu Liu, Manman Xu, Qiaojian Zou, Yijia Zhou, Hua Huang, Yuwen Pan, Wei Wang, Shuzhong Yao

**Affiliations:** 1 Department of Obstetrics and Gynecology, the First Affiliated Hospital, Sun Yat-sen University, 510080 Guangzhou, Guangdong China; 2 Department of Obstetrics and Gynecology, Peking University Third Hospital, 100191 Beijing, China

**Keywords:** Cervical cancer, Metastasis

## Abstract

Epithelial-mesenchymal transition (EMT) is an essential step to drive the metastatic cascade to lymph nodes (LNs) in cervical cancer cells. However, few of them metastasize successfully partially due to increased susceptibility to immunosurveillance conferred by EMT. The precise mechanisms of cancer cells orchestrate EMT and immune evasion remain largely unexplored. In this study, we identified a lncRNA termed lymph node metastasis associated suppressor (LNMAS), which was downregulated in LN-positive cervical cancer patients and correlated with LN metastasis and prognosis. Functionally, LNMAS suppressed cervical cancer cells metastasis in vitro and in vivo. Mechanistically, LNMAS exerts its metastasis suppressive activity by competitively interacting with HMGB1 and abrogating the chromatin accessibility of TWIST1 and STC1, inhibiting TWIST1-mediated partial EMT and STC1-dependent immune escape from macrophage phagocytosis. We further demonstrated that the CpG sites in the promoter region of LNMAS was hypermethylated and contributed to the downregulation of LNMAS. Taken together, our results reveal the essential role of LNMAS in the LN metastasis of cervical cancer and provide mechanistic insights into the regulation of LNMAS in EMT and immune evasion.

## Introduction

Cervical cancer (CCa) is one of the most common gynecologic cancers, especially in regions with low or medium Human Development Index [1]. Lymph node (LN) metastasis is the main metastatic way in CCa and contributes to a much worse prognosis [2, 3]. Considering its significant influence on prognosis, the 2018 FIGO staging has regarded LN metastasis alone as a new criterion for stage IIIC [4]. However, the current treatment options for LN-positive (LNpos) CCa patients are limited and up for debate. Thus, a more comprehensive understanding of the underlying molecular mechanism in LN metastasis is critical to provide potential therapeutic targets and improve prognosis.

LN metastasis is a multistep process with complex biological mechanisms participated, including lymphangiogenesis, cancer cells dissemination to lymphatic vessels, drainage of cancer cells into sentinel LNs, and settlement and colonization of cancer cells in LNs [5]. Epithelial-mesenchymal transition (EMT) is an essential step to drive the metastatic cascade [6, 7]. During EMT process, epithelial cells lose polarity and gain invasive properties to disseminate to lymphatic vessels, but few of them metastasize successfully, partially due to increased susceptibility to metastasis-specific immunosurveillance conferred by EMT [8]. Researches have shown that EMT-activated cancer cells can take advantage of immune-checkpoint molecules (ICMs), such as CD47, to achieve immune escape by delivering a “don’t eat me” signal to macrophages in breast cancer [9, 10]. As some preclinical studies and clinical data have demonstrated the promise of targeting phagocytosis checkpoints [11, 12], macrophage phagocytosis checkpoints, including CD47, CD24, stanniocalcin 1 (STC1), are potential targets for cancer immunotherapy in patients with advanced cancers [13, 14]. Therefore, elucidating the regulatory mechanisms that cancer cells orchestrate EMT and immune evasion may provide valuable evidence for the treatment of CCa patients.

Accumulating evidence has highlighted that long non-coding RNAs (lncRNAs), a large class of RNA transcripts over 200 nucleotides in length which lack protein-coding capability, play an important role in cancer metastasis [15]. LncRNAs, such as LINC00941 [16] and SMASR [17], exert promotive or suppressive effects in EMT. LncRNA NKILA [18] and ALAL-1 [19] were also reported to have regulatory functions in cancer immune evasion. However, the role of lncRNAs in coordinating EMT and immune evasion remains to be elucidated in CCa.

In this study, we have discovered a LN metastasis associated lncRNA, LN metastasis associated suppressor (LNMAS), by systematic screening in CCa samples. We validated the expression and biologic function of LNMAS in CCa. Furthermore, we demonstrated that LNMAS interacted with high mobility group box 1 (HMGB1) and abrogated the chromatin accessibility of TWIST1 and STC1, exerting its metastasis suppressive activity by inhibiting TWIST1-mediated EMT and STC1-dependent immune escape from macrophage phagocytosis. Meanwhile, we also found that the expression of LNMAS was regulated by CpG methylation in the promoter region. Taken together, these findings uncover novel insights into the mechanism of LN metastasis and provide potential therapeutic targets in CCa.

## Results

### LNMAS correlates with LN metastasis in cervical cancer

**Fig. 1:**
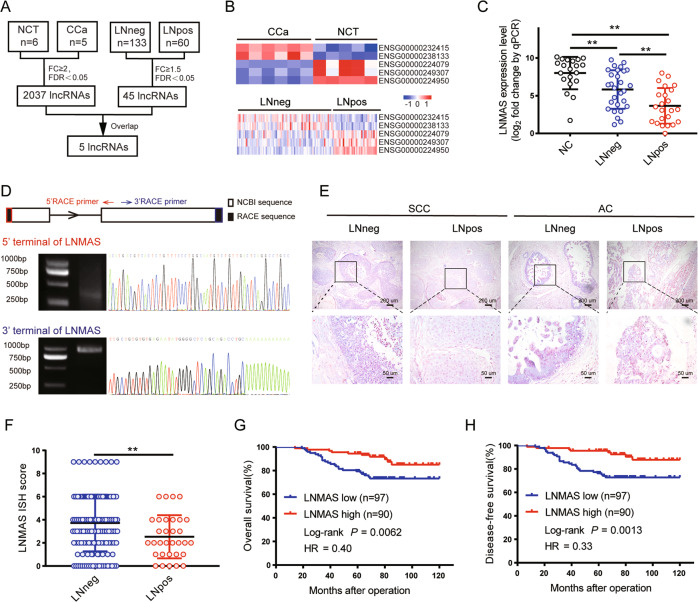
Downregulation of LNMAS is associated with LN metastasis and poor prognosis for cervical cancer. **A** Schematic representation of systematic screening for LN metastasis associated lncRNAs in cervical cancer. **B** Heatmaps for the overlapped dysregulated lncRNAs. **C** QPCR analysis of LNMAS expression in an 82-case cohort of freshly collected human cervical cancer samples and normal cervix tissues. Statistical significance was assessed by ANOVA test. **D** Representative image of agarose gel electrophoresis and sequencing of 5'-RACE and 3'-RACE products of LNMAS. Representative ISH images (**E**) and ISH socres (**F**) of LNMAS expression (blue) in the paraffin-embedded tumor sections of cervical cancer with or without LN metastasis (*n* = 187). Statistical significance was assessed by Student’s *t* test. Kaplan–Meier curves for OS (**G**) and DFS (**H**) of cervical cancer patients with low vs. high expression of LNMAS. Statistical significance was assessed by log-rank test. Data are presented as mean ± SD, *P < 0.05, **P < 0.01.

To identify the dysregulated lncRNAs that contribute to LN metastasis in CCa, a systematic screening was conducted in a local lncRNA microarray dataset and the Cancer Genome Atlas (TCGA) dataset (Fig. 1A). The lncRNA expression profiles were obtained from six normal cervical tissues (NCT) and five CCa tissues by lncRNA microarray. 2037 lncRNAs were differentially expressed by more than 2-fold change, 973 upregulated and 1064 downregulated. We further analyzed the dysregulated lncRNAs in 133 CCa patients without LN metastasis (LNneg) and 60 CCa patients with LN metastasis (LNpos) from TCGA. 45 lncRNAs were differentially expressed by more than 1.5-fold change, 14 upregulated and 31 downregulated. Five lncRNAs were consistently dysregulated in both datasets (Fig. 1B), including ENSG00000238133, ENSG00000224079, ENSG00000249307 and ENSG00000224950, which were reported to correlate with human cancer progression [20–23]. Additionally, ENSG00000232415, termed LNMAS here, was significantly downregulated in the LNpos group, as determined by qPCR (Fig. 1C). LNMAS is located at human chromosome 7q11.23 (NCBI: XR_001745243.1). The full-length of LNMAS was identified by the 5′ and 3′ rapid amplification of cDNA ends (RACE). We obtained 50 and 49 bp unannotated sequence at the 5' and 3' end respectively, with a polyA structure at the 3' end (Fig. 1D). Bioinformatic prediction and protein-coding assays confirmed that LNMAS lacks protein-coding capability (Supplementary Fig. S1A, B).

We performed in situ hybridization (ISH) on 187 cases of CCa specimens to further explore the clinical role of LNMAS. The results showed that the LNneg cases had higher ISH scores than LNpos cases (Fig. 1E, F). According to the ISH score of LNMAS, we divided patients into two groups: low-LNMAS group (ISH score ≤4) and high-LNMAS group (ISH score >4). We analyzed the correlation between the expression level of LNMAS and clinicopathological characteristics. A lower level of LNMAS significantly correlated with tumor size (*P* = 0.028) and LN metastasis (*P* = 0.036) (Table 1). Importantly, Kaplan–Meier survival curves showed that low LNMAS expression was associated with decreased overall survival (OS) and disease-free survival (DFS) (Fig. 1G, H). Multivariate cox proportional hazards analyses showed that LNMAS expression was an independent prognostic factor for OS and DFS (Supplementary Fig. S2A). Moreover, analyses of TCGA dataset showed that LNMAS was commonly reduced in human cancers and predicted prognosis in CCa (Supplementary Fig. S2B, C), which further suggested that LNMAS may play a suppressive role in the progression of human cancers.

**Table 1:** LNMAS levels and clinicopathological features in 187 cervical cancer patients.

Characteristics	Total 187	LNMAS		*P*-value
		Low (97)	High (90)	
Age (year)				0.25
≤40	45	20	25	
>40	142	77	65	
Pathologic types				0.52
Squamous carcinoma	148	75	73	
Adenosquamous and adenocarcinoma	39	22	17	
FIGO stage (2009)				0.70
I	158	81	77	
II	29	16	13	
Differentiation				0.93
G1	18	10	8	
G2	61	32	29	
G3	108	55	53	
Tumor size				0.028
≤4 cm	152	73	79	
>4 cm	35	24	11	
Lymphovascular invasion				0.99
No	162	84	78	
Yes	25	13	12	
Stromal invasion				0.17
≤1/2	111	53	58	
>1/2	76	44	32	
Parametrial infiltration				0.93
No	181	94	87	
Yes	6	3	3	
Vaginal involvement				0.12
No	181	92	89	
Yes	6	5	1	
Lymph node metastasis				0.036
No	155	75	80	
Yes	32	22	10	

### LNMAS inhibits cervical cancer metastasis in vitro and in vivo

**Fig. 2:**
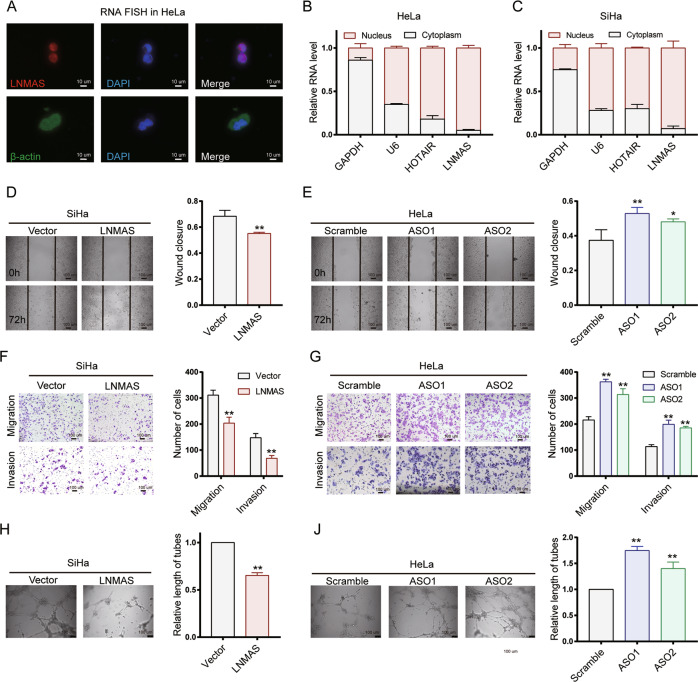
LNMAS inhibits cervical cancer cells metastasis and lymphangiogenesis in vitro. **A** Representative RNA FISH images of LNMAS and β-actin in HeLa. Subcellular fractionation assays and qPCR analyses of LNMAS expression in the nucleus and cytoplasm of HeLa (**B**) and SiHa (**C**). Representative images of wound healing assays using SiHa and HeLa after overexpression (**D**) or knockdown (**E**) of LNMAS. Representative images of transwell assays using SiHa and HeLa after overexpression (**F**) or knockdown (**G**) of LNMAS. Representative images of HLEC tube formation assays using the conditioned media from SiHa and HeLa after overexpression (**H**) or knockdown (**J**) of LNMAS. Statistical significance was assessed by Student’s *t* test in **D**, **F**, **H** and one-way ANOVA test in **E**, **G**, **J**. Data are presented as mean ± SD, *n* = 3, *P < 0.05, **P < 0.01.

To determine the potential role of LNMAS in LN metastasis, gain- and loss-of function experiments were performed. Two typical CCa cell lines, SiHa and HeLa, were selected for further experiments. SiHa and HeLa showed a lower expression level of LNMAS than the normal cervical epithelial cell H8 (Supplementary Fig. S3A). According to fluorescence in situ hybridization (FISH) (Fig. 2A, Supplementary Fig. S3B), LNMAS was primarily located in the nucleus, which was confirmed by subcellular fractionation assays (Fig. 2B, C). We manipulated the expression of LNMAS with LNMAS-overexpression lentiviral plasmids or LNMAS-specific antisense oligos (ASOs), which were confirmed by qPCR (Supplementary Fig. S3C).

The results of transwell and wound healing assays showed that overexpression of LNMAS decreased the invasion and migration ability of SiHa and HeLa cells (Fig. 2D, F, and Supplementary Fig. S3D, F), whereas knockdown of LNMAS significantly increased the invasion and migration ability (Fig. 2E, G, and Supplementary Fig. S3E, G). Lymphangiogenesis is a crucial step for LN metastasis, we further investigated the effects of LNMAS on lymphatic tube formation. Compared with the corresponding control groups, culture supernatants from LNMAS overexpressed cells significantly inhibited human lymphatic endothelial cells (HLEC) tube formation, while culture supernatants from LNMAS silenced cells showed the opposite results (Fig. 2H, J). As the clinicopathological analysis showed that LNMAS significantly correlated with tumor size, we performed CCK8 and colony formation assays to investigate the effects of LNMAS on cancer cells proliferation and tumor growth. However, colony formation assays showed that LNMAS knockdown or overexpression had no significant effects on the proliferative capacity (Supplementary Fig. S4A, B), consistent with the results of CCK8 assays (Supplementary Fig. S4C, D). Taken together, LNMAS exerted a suppressive impact on CCa cells metastasis and lymphangiogenesis, other than cancer cells proliferation, in vitro.

**Fig. 3:**
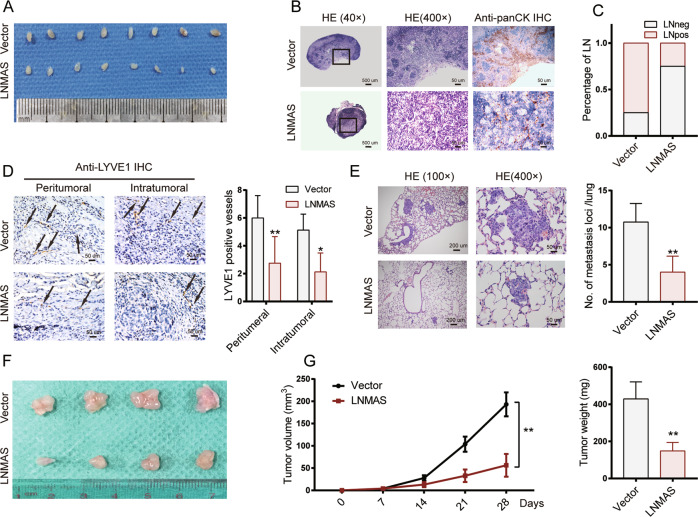
LNMAS inhibits cervical cancer cells metastasis and tumor growth in vivo. **A** Representative images of popliteal LNs in the indicated mice group. **B** Representative images of HE and IHC staining with anti-pan-keratin antibody in the indicated mice group. **C** The popliteal LN metastasis rate in the indicated mice group. **D** Representative IHC images of peritumoral (*n* = 8) and intratumoral (*n* = 8) lymphatic vessels stained by anti-LYVE-1 in the primary tumors resected from footpads in the indicated mice group. **E** Representative HE images of lung metastasis in the indicated mice group (*n* = 4). **F** Representative images of subcutaneous xenografts in the indicated mice group. **G** Tumor growth curves (*n* = 4) and the weight of tumors upon sacrifice in the indicated mice group (*n* = 4). Statistical significance was assessed by Student’s *t* test. Data are presented as mean ± SD, *P < 0.05, **P < 0.01.

We further conducted animal experiments to determine the effects of LNMAS on LN metastasis in vivo. An in vivo popliteal LN metastasis model was employed. Results showed that LNMAS overexpressed group had smaller volumes of popliteal LNs than the control group (Fig. 3A). As determined by hematoxylin and eosin staining (HE) and immunohistochemistry (IHC), LNMAS overexpression reduced the LN metastasis rate (Fig. 3B, C). The density of microlymphatic vessels also decreased (Fig. 3D). Tail vein injection and subcutaneous tumorigenicity assays were also employed to illustrate the effects of LNMAS on CCa metastasis and tumor growth. Overexpression of LNMAS significantly decreased the number of lung metastasis (Fig. 3E) and the volume of subcutaneous tumor (Fig. 3F, G). Collectively, LNMAS inhibited CCa cells metastasis, lymphangiogenesis and tumor growth in vivo.

### LNMAS attenuates EMT and immune escape from macrophage phagocytosis

**Fig. 4:**
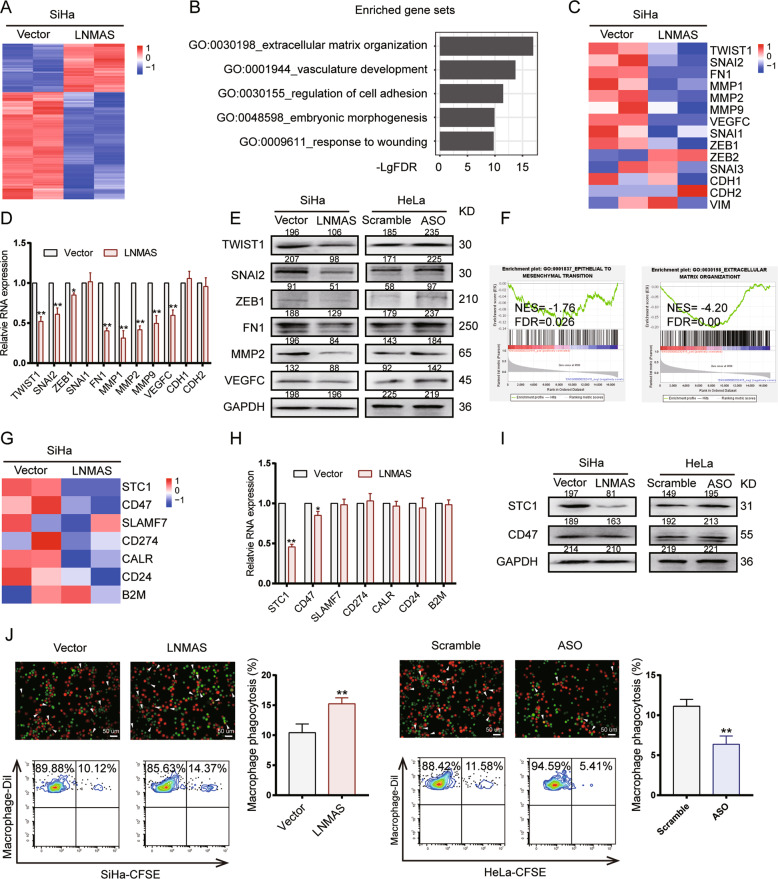
LNMAS attenuates EMT and evasion of macrophage phagocytosis. **A** Unsupervised hierarchical clustering of the differentially expressed genes in LNMAS overexpressed SiHa and corresponding control cells. **B** The gene annotation results of DEGs by Metascape. **C** The RNA expression profile of EMT-associated genes in the RNA-seq data. **D** The relative mRNA expression of EMT-associated genes qualified by qPCR. **E** The protein expression of EMT-associated genes qualified by western blot in the indicated group and the grey value (0–255) were displayed. **F** GSEA results of EMT and extracellular matrix organization in TCGA dataset grouped by LNMAS expression. **G** The RNA expression profile of several immune checkpoints in the RNA-seq data. **H** The relative mRNA expression of immune checkpoints qualified by qPCR. **I** The protein expression of immune checkpoints qualified by western blot in the indicated group and the grey value (0–255) were displayed. **J** The macrophage phagocytosis results detected by microscope and flow cytometry in the indicated group. Statistical significance was assessed by Student’s *t* test. Data are presented as mean ± SD, *n* = 3, *P < 0.05, **P < 0.01.

To identify the potential targets of LNMAS, RNA-seq was performed. We obtained 430 differentially expressed genes (DEGs) by more than 2-fold change, 91 upregulated and 339 downregulated (Fig. 4A). Gene annotation analyses showed that the DEGs were significantly enriched in several metastasis-related pathways, including extracellular matrix organization, vasculature development and regulation of cell adhesion (Fig. 4B). EMT is a critical step in cancer cells metastasis by regulating extracellular matrix organization and cell adhesion [6]. Several EMT-associated genes were significantly dysregulated according to the RNA-seq data, including FN1, TWIST1 and SNAI2 (Fig. 4C). We further validated the expression of EMT-associated genes by qPCR and western blot (Fig. 4D, E, Supplementary Fig. S5G). The results showed that LNMAS attenuated the expression of mesenchymal markers, while the epithelial markers remained largely unchanged, suggesting that LNMAS may inhibit CCa metastasis by abrogating the partial EMT phenotype. This result was further confirmed by gene set enrichment analysis (GSEA) of TCGA dataset, where the expression level of LNMAS negatively correlated with EMT and extracellular matrix organization (Fig. 4F).

As mentioned above, LNMAS inhibited tumor growth in vivo, while it had no significant effects on CCa cells proliferation in vitro, indicating that the tumor immune microenvironment may participate in the role of LNMAS. Cancer cells were reported to take advantage of ICMs, such as PDL1 and CD47, to achieve immune evasion [24]. To validate our hypotheses, we analyzed the expression of several ubiquitous ICMs in the RNA-seq data (Fig. 4G). The results showed that STC1, an ICM reported to participate in the macrophage phagocytosis, was significantly downregulated in LNMAS overexpressed cells. Another macrophage phagocytosis checkpoint molecule, CD47, was also downregulated slightly. We further validated their expression by qPCR and western blot (Fig. 4H, I). To confirm the effects of LNMAS on macrophage phagocytosis, in vitro macrophage phagocytosis assays were performed. The results showed that macrophage devoured more LNMAS overexpressed cells and less LNMAS silenced cells, compared to their corresponding control cells (Fig. 4J). Taken together, LNMAS inhibited CCa metastasis by attenuating EMT and immune escape from macrophage phagocytosis.

### TWIST1 and STC1 are the direct targets of LNMAS

**Fig. 5:**
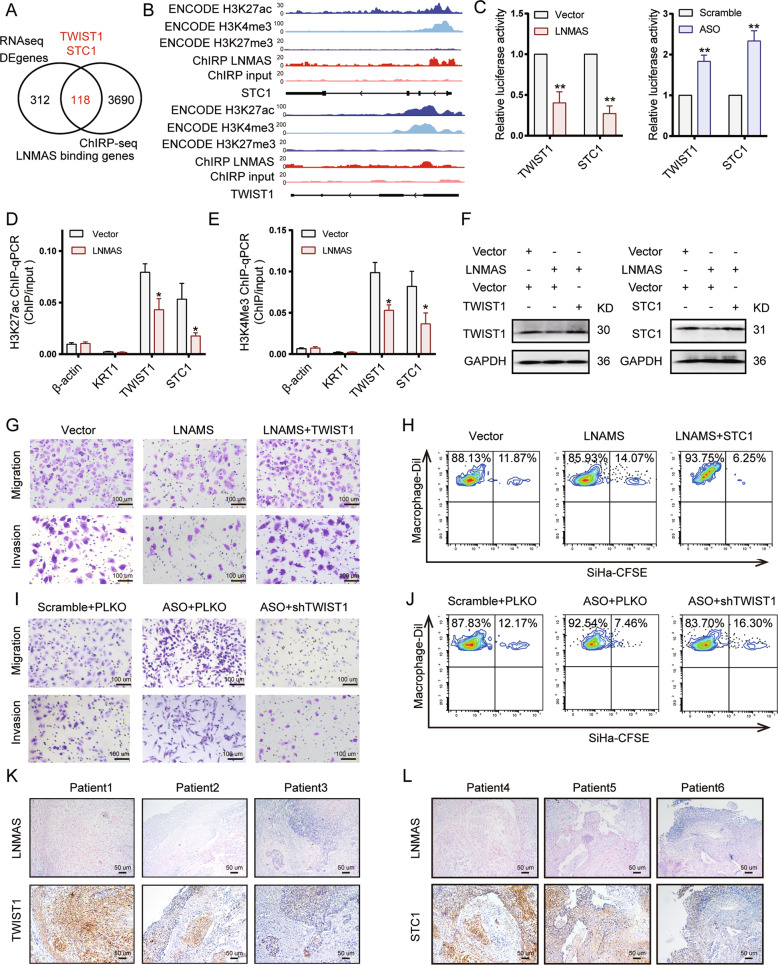
TWIST1 and STC1 are the direct targets of LNMAS. **A** The venn plot of RNA-seq DEGs and LNMAS-binding genes detected by ChIRP-seq. **B** The LNMAS-binding locus, H3K27ac, H3K4me3 and H3K27me3 modification sites in the promoters of STC1 and TWIST1. **C** The relative luciferase activity in SiHa and HeLa of the promoters of TWIST1 and STC1, respectively. **D**, **E** ChIP-qPCR analysis of H3K27ac and H3K4me3 occupancy in the promoters of β-actin, KRT1, TWIST1 and STC1 in the indicated group, respectively. KRT1 was used as the negative control. **F** The protein expression of TWIST1 and STC1 in the indicated group. **G**, **I** The representative images of transwell assays in the indicated group. PLKO: the shRNA vector. **H**, **J** The representative images of in vitro macrophage phagocytosis assays in the indicated group. **K**, **L** The representative ISH images of LNMAS and IHC images of STC1 in clinical cervical cancer tissues. Statistical significance was assessed by Student’s *t* test. Data are presented as mean ± SD, *n* = 3, *P < 0.05, **P < 0.01.

Nucleus enriched lncRNAs were reported to form complexes with chromatins and proteins to regulate gene expression [25]. To find the direct target of LNMAS, we performed ChIRP-seq to detect the DNA regions that LNMAS can bind and overlapped the LNMAS-binding genes with the DEGs identified by RNA-seq (Fig. 5A, Supplementary Fig. S5A). Among the 118 reduplicate genes were TWIST1 and STC1, which was confirmed by ChIRP-qPCR (Supplementary Fig. S5B). We speculated that LNMAS may attenuate EMT and immune evasion by directly targeting TWIST1 and STC1, respectively. We found that the genomic binding regions of LNMAS were located at the promoters of TWIST1 and STC1, where histone modifications, including H3K4me3 and H3K27ac, were annotated by the ENCODE project (Fig. 5B, Supplementary Fig. S6A). H3K4me3 and H3K27ac are important markers of chromatin accessibility and regulate downstream gene transcription. Thus, we examined the transcriptional activity and found that LNMAS significantly reduced the transcriptional activity of the promoters of TWIST1 and STC1 (Fig. 5C). We further conducted ChIP-qPCR to investigate the effects of LNMAS on histone modifications of target genes. The results showed that H3K4me3 and H3K27ac modifications were less enriched in LNMAS overexpressed cells (Fig. 5D, E), while the enrichment of H3K27me3, a repressive marker of transcription, increased (Supplementary Fig. S6B). These results indicated that LNMAS may modulate the transcription of target genes by histone modification regulation.

Furthermore, we restored the expression of TWIST1 and STC1 in LNMAS overexpressed cells (Fig. 5F) and determined their effects on cancer cells metastasis and macrophage phagocytosis, respectively. Restoration of TWIST1 partially rescued the migration and invasion capability (Fig. 5G) and restoration of STC1 decreased the phagocytosis capability of macrophages (Fig. 5H). On the contrary, we knocked down TWIST1 and STC1 in LNMAS silenced cells (Supplementary Fig. S6C), the migration and invasion capability decreased (Fig. 5I) and the phagocytosis capability of macrophages increased (Fig. 5J). These results indicated that the regulatory effects of LNMAS relied on TWIST1 and STC1. We also observed a negative correlation between the expression of LNMAS and TWIST1 (Fig. 5K), LNMAS and STC1 (Fig. 5L) in clinical CCa samples.

### LNMAS directly interacts with HMGB1

**Fig. 6:**
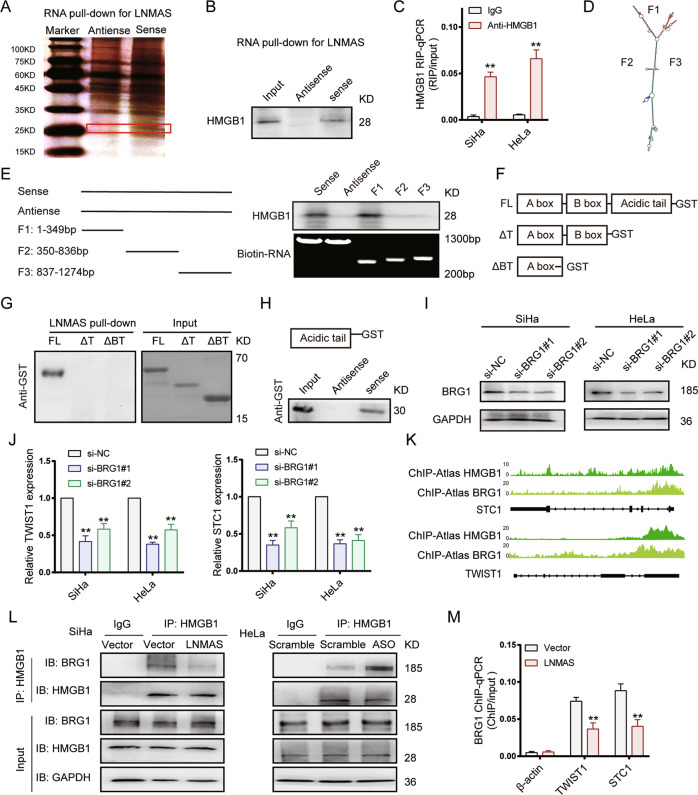
LNMAS perturbs HMGB1-BRG1 interaction to modulate target genes expression. **A** The silver staining image of proteins pulled down by LNMAS sense and antisense RNAs. **B** Detection of HMGB1 by western blot in the proteins pulled down by LNMAS sense and antisense RNAs. **C** The retrieved LNMAS by IgG and Anti-HMGB1 using RIP assays in SiHa and HeLa. Statistical significance was assessed by Student’s *t* test. **D** The secondary structure of LNMAS predicted by RNAfold. **E** Serial deletions of LNMAS were used in the RNA pull-down assays to identify the core regions of LNMAS for the physical interaction with HMGB1. **F**, **G** A series of truncated recombinant HMGB1 proteins with GST tag were used in the in vitro binding assays to identify the core domain of HMGB1 for the physical interaction with LNMAS. **H** Western blot of acidic tail of HMGB1 with GST tag retrieved by LNMAS sense and antisense RNAs. **I** Western blot of BRG1 in the indicated groups. **J** The mRNA expression of TWIST1 qualified by qPCR in SiHa (*n* = 3) and HeLa (*n* = 3) with BRG1 knockdown (left panel); The mRNA expression of STC1 qualified by qPCR in SiHa (*n* = 3) and HeLa (*n* = 3) with BRG1 knockdown (left panel). Statistical significance was assessed by one-way ANOVA test. **K** HMGB1 and BRG1 occupancy in the promoters of TWIST1 and STC1 annotated by ChIP-Atlas. **L** Western blot of BRG1 retrieved by HMGB1 using CoIP assays with LNMAS overexpression (left panel) or knockdown (right panel). **M** QPCR of TWIST1 (*n* = 3) and STC1 (*n* = 3) retrieved by BRG1 using ChIP assays in the indicated group. Statistical significance was assessed by Student’s *t* test. *P < 0.05, **P < 0.01.

To further investigate the mechanisms that LNMAS regulate histone modification, we subsequently conducted RNA pull-down assays using in vitro transcribed biotinylated LNMAS to determine the proteins that LNMAS may interact. An obvious band was observed between 25 and 35 kDa in the proteins retrieved by LNMAS (Fig. 6A). HMGB1 was determined as one of the most abundant proteins that LNMAS interacted via mass spectrometry (MS) (Fig. 6B). We further performed RIP assays and found that LNMAS directly interacted with HMGB1 (Fig. 6C). To identify the specific region of LNMAS that may interact with HMGB1, a deletion-mapping system based on the predicted secondary structure of LNMAS (Fig. 6D) was used. The results showed that 5′ region of the LNMAS (1–349 nt) was required for direct interaction with HMGB1 (Fig. 6E).

HMGB1 is mainly localized in the nucleus, where it interacts with DNA and regulates histone modification [26, 27]. To assess which domain of HMGB1 regulated its binding to LNMAS, we employed a deletion-mapping approach. A series of truncated recombinant HMGB1 proteins with GST tag were used in the in vitro binding assays (Fig. 6F). The results showed that deletion of the acidic tail domain abolished the interaction with LNMAS (Fig. 6G), consistent with the in vitro binding of acidic tail domain and LNMAS (Fig. 6H). Collectively, our findings demonstrated that LNMAS interacted with the acidic tail domain of HMGB1 through its 1–349 nt sequence.

### LNMAS perturbs HMGB1-BRG1 interaction to modulate target genes expression

HMGB1 was reported to regulate histone modification with the chromatin remodeling complexes, such as SWI/SNF complex [27]. BRG1, the core subunit of SWI/SNF complex to regulate histone modification and gene transcription, can interact with HMGB1 to promote prostate cancer metastasis [28]. Research has shown that BRG1 activates TWIST1 transcription by modulating H3K4me3 modification [29]. Thus, we validated the regulatory effect of BRG1 on the target genes of LNMAS (TWIST1 and STC1). The results showed that RNA expression of TWIST1 and STC1 significantly decreased in BRG1 silenced CCa cells (Fig. 6I, J).

According to the ChIP-seq data in ChIP-Atlas [30] and our ChIRP-seq results, the binding location of LNMAS, HMGB1 and BRG1 partially overlapped in the promoters of TWIST1 and STC1 (Figs. 5B, 6K). As mentioned above, LNMAS directly interacted with the acidic tail domain of HMGB1, which regulated the binding affinity to other proteins and DNA bending [31]. Thus, we speculated that LNMAS perturbs HMGB1-BRG1 interaction to inhibit the histone modification in the promoters of TWIST1 and STC1. To test this hypothesis, the interaction between HMGB1 and BRG1 was examined by co-immunoprecipitation (CoIP) assays, in the presence or absence of LNMAS. LNMAS knockdown could enhance the interaction between HMGB1 and BRG1, while LNMAS overexpression attenuated their interaction (Fig. 6L). Furthermore, LNMAS overexpression reduced BRG1, but not HMGB1, occupancy at TWIST1 and STC1 promoters (Fig. 6M, Supplementary Fig. S6D). Collectively, these results demonstrated that LNMAS formed a lncRNA-HMGB1 complex in TWIST1 and STC1 promoters and abrogated their expression of by perturbing HMGB1-BRG1 interaction.

### LNMAS expression is determined by the DNA methylation status in the promoter

**Fig. 7:**
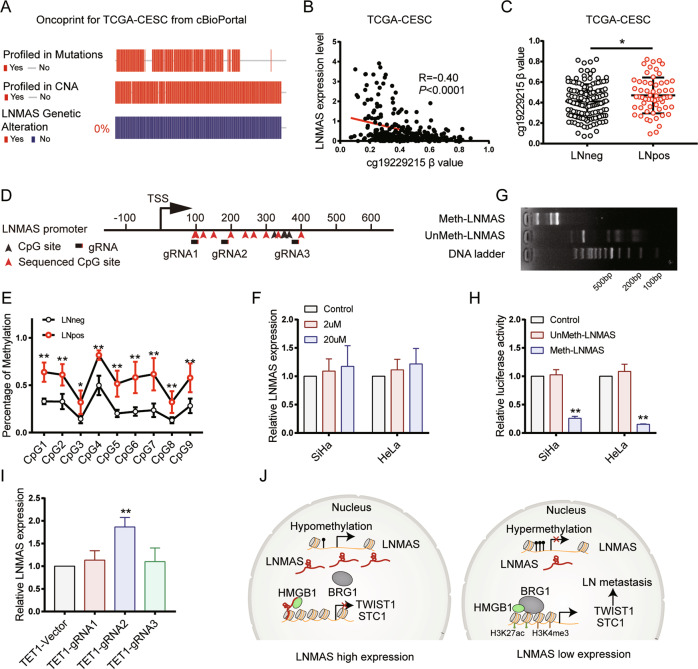
LNMAS expression is determined by the DNA methylation status in the promoter. **A** The oncoprint results for genomic alteration of LNMAS using cbioportal. **B** The pearson correlation of LNMAS expression and CpG methylation from TCGA dataset. **C** The CpG methylation level of LNMAS promoter in LNpos (*n* = 60) and LNneg (*n* = 133) group in TCGA dataset. Statistical significance was assessed by Student’s *t* test. **D** Schematic representation of the genomic sequence of LNMAS promoter. **E** The pyrosequencing of LNMAS promoter in LNneg (*n* = 5) and LNpos (*n* = 5) cervical samples. Statistical significance was assessed by Student’s *t* test. **F** The LNMAS expression qualified by qPCR in cervical cancer cells treated with DAC. **G** In vitro DNA methylation status of LNMAS promoters was confirmed by Hpall restriction enzyme. **H** The relative luciferase activity of methylated and unmethylated LNMAS promoters in SiHa (*n* = 3) and HeLa (*n* = 3). Statistical significance was assessed by Student’s *t* test. **I** The expression of LNMAS qualified by qPCR in SiHa treated with DNA demethylation plasmids targeting LNMAS. Statistical significance was assessed by one-way ANOVA test. **J** Illustrative model showing the proposed mechanism by which downregulation of LNMAS orchestrates EMT and evasion of macrophage phagocytosis to promote LN metastasis. *P < 0.05, **P < 0.01.

To further investigate the mechanisms underlying the aberrant expression of LNMAS, we first analyzed the genomic alteration of LNMAS in CCa via the cbioportal website based on TCGA database. The results showed that no genomic alteration was found in the genomic sequence of LNMAS (Fig. 7A), but the expression of LNMAS negatively correlated with DNA methylation level of cg19229215, a CpG site in its promoter (Fig. 7B). Besides, compared with LNneg patients, a higher methylation level was found in LNpos patients in TCGA (Fig. 7C). To further clarify the relationship between CpG methylation and LN metastasis, we performed pyrosequencing in 5 LNpos and 5 LNneg CCa samples collected in our center. The results showed that the CpG methylation levels in LNMAS promoter were hypermethylated in LNpos patients (Fig. 7D, E). Thus, we speculated that hypermethylation of LNMAS promoter contributed to the downregulation of LNMAS, thereby promoting the occurrence of LN metastasis.

We treated CCa cells with decitabine (DAC), a DNA methyltransferase inhibitor, to validate our hypothesis. The results showed that the expression of LNMAS slightly increased, without statistical significance (Fig. 7F). An in vitro methylation assay was conducted to further demonstrate the influence of CpG methylation on the expression of LNMAS. We cloned the LNMAS promoter sequence into pGL3 basic vector and found that the unmethylated LNMAS promoter (unMeth-LNMAS) led to a significantly higher reporter activity compared with the methylated version (Meth-LNMAS) (Fig. 7G, H). The results demonstrated that LNMAS is directly regulated by the CpG methylation in its promoter. Recently, CRISPR-dCas9 system was employed to manipulate CpG methylation directly and efficiently [32–34]. We designed 3 gRNAs in LNMAS promoter and cloned them into dCas9-TET1-CD vector to edit the CpG methylation (Fig. 7D). The results showed that gRNA2 can significantly affect the expression of LNMAS, but not gRNA1 and gRNA3 (Fig. 7I). Furthermore, demethylating LNMAS by gRNA2 inhibited the partial EMT phenotype of CCa cells and restored the macrophage phagocytosis in vitro (Supplementary Fig. S7A–C). Collectively, these results demonstrated that LNMAS expression is determined by the DNA methylation status in its promoter and methylation editing based on CRISPR-dCas9 system is a potential tool for target therapy.

## Discussion

LN metastasis confers a worse prognosis in CCa patients, but the current treatment options are limited and up for debate. Thus, a better understanding of the underlying molecular mechanisms in LN metastasis is critical to provide potential therapeutic targets. Herein, we investigated the critical role of LNMAS in LN metastasis of CCa. We demonstrated that LNMAS inhibited TWIST1-mediated EMT and STC1-dependent immune evasion via attenuating the interaction of HMGB1 and BRG1. The downregulation of LNMAS determined by DNA hypermethylation promoted LN metastasis.

EMT is an essential step to drive the metastatic cascade in CCa [35]. TWIST1, an important EMT-TF, was reported to participate in the progression of CCa (Supplementary Fig. S5C, D) [36]. Typical EMT is characterized by the downregulation of epithelial marker E-cadherin and upregulation of mesenchymal marker N-cadherin, mainly driven by SNAI1. In our study, we identified TWIST1 as a direct target of LNMAS. Obvious changes in SNAI2, FN1, MMPs, but not epithelial markers, were observed. This partial EMT phenotype is known for more malignant behavior of cancer cells [37], consistent with the results found in glioblastoma, where TWIST1 did not generate an E- to N-cadherin “switch” in GBM cell lines [38]. These findings indicate that the EMT phenotypes are context dependent [39] and CCa cells may not go through a full EMT, but rather adopt some qualities of mesenchymal cells and keep some epithelial characteristics during LN metastasis. Furthermore, the EMT phenotype often accompanied with increased lymphangiogenesis [40, 41]. We observed that LNMAS exerted an inhibitory effect on lymphangiogenesis in vitro and in vivo (Fig. 2H, J, D) by reducing VEGFC derived from cancer cells (Fig. 4C–E). TWIST1, the direct target of LNMAS and a well-known inducer of EMT, was probably the intermediator of LNMAS and VEGFC. Researches have shown that TWIST1 dependent VEGFC expression promoted the progression of cholangiocarcinoma [42]. However, the molecular mechanisms of TWIST1 regulating VEGFC are poorly explored. Whether VEGFC is the direct target of TWIST1 or a by-product of EMT remains unknown. In the context of CCa, the relationship between LNMAS, TWIST1 and VEGFC requires further investigation.

Cancer cells must avoid attack by immune cells successfully to survive and metastasize to LNs. Macrophages are one of the most abundant immune cells infiltrating in the microenvironment of CCa [43]. CCa cells may take advantage of ICMs, such as CD47, to escape from the phagocytosis and antigens presentation mediated by macrophages [44]. In this study, we found that STC1, another phagocytosis checkpoint [14], was a direct target of LNMAS and contributed to the immune escape from macrophages. STC1 is commonly upregulated in various cancer types and correlates with poor prognosis [14]. STC1 also confers a worse prognosis in CCa in TCGA (Supplementary Fig. S5E, F), but some studies also have reported that STC1 can inhibit proliferation and promote apoptosis in CCa cells [45, 46]. The complex role of STC1 in CCa progression requires further research.

Emerging evidence has confirmed that nucleus localized lncRNAs function as scaffolds or decoys to regulate histone modification [47]. Our results revealed that LNMAS repelled BRG1 from the promoters of TWIST1 and STC1 through directly binding HMGB1, influencing histone modification and transcription inhibition. BRG1 is a catalytic subunit of the SWI/SNF chromatin remodeling complex, with key roles in modulating histone modification and chromatin accessibility [48]. HMGB1 has been reported to interact with BRG1 to promote prostate cancer metastasis [28], and it is a potential therapeutic target to abrogate their interaction. Our results indicated that LNMAS attenuated their interaction in the promoters of TWIST1 and STC1, making LNMAS a potential gene-specific intervention target in CCa patients. To develop lncRNA-based therapeutics for LNMAS intervention are promising but challenging. None of the lncRNA-based therapies, such as ASOs and siRNAs, have been translated into clinic use for the low specificity, hurdles of immunogenicity, and nonspecific delivery [49]. Recently an innovative RNA-based strategy was proposed by designing a HOTAIR deletion mutant form with the putative Snail-binding domain but depleted of the EZH2-binding domain [50]. The mutant HOTAIR competitively bound Snail and impaired the EMT phenotype. This method provides new insights into lncRNA-based therapeutics and may be a potential strategy for LNMAS-targeting intervention, which requires further exploration.

To our interest, we found that LNMAS suppresses growth of CCa cells in vivo but not in vitro (Fig. 3F, G, Supplementary Fig. S4A–D). The proliferation and metastasis of cancer cells remained controversial under certain circumstance. The highly LN metastatic cell line established by Choong-Kun Lee et al. [51] displayed slower growth in the primary tumor. EMT-inducing factors were shown to exert no or negative effects on cancer cells proliferation [52, 53], among which ZEB1, induced by RP11, showed no significant effects in proliferation in colorectal cancer [54]. Likewise, TWIST1 was also reported to promote proliferation of early metastatic colonies, but not primary tumor in squamous cell carcinoma [55]. We speculated that cancer cells may give up rapid proliferation for the priority to dissemination and metastasize. CCa cells may choose metastasis rather than proliferation by LNMAS depletion and TWIST1 induction. However, tumor growth in vivo was determined not only by the intrinsic capability, but also by the tumor microenvironment [56]. Eliminating cancer cells by immune cells gave rise to not only failure to metastasis, but also reduction of primary tumor [57]. Inhibition of STC1 by LNMAS increased macrophage phagocytosis capability and that may be the underlying reason why LNMAS inhibited the growth of CCa cells in vivo but not in vitro.

Another important finding in this study was that DNA hypermethylation in the promoter determined the aberrant expression of LNMAS in CCa patients. Studies have found most gynecological malignancies, including CCa, have DNA hypermethylation, which inactivates tumor suppressor genes [58]. DAC is a highly effective inhibitor of DNA methyltransferase and approved for the treatment of myelodysplastic syndrome (MDS). When we treated CCa cells with DAC, the expression of LNMAS increased without statistics significance. The development of CRISPR gene editing technology has brought new possibilities for DNA methylation editing in specific sites [32–34]. We used a dCas9-TET1 system reported in the literature and restored the expression of LNMAS in CCa cells with the partial EMT phenotype and escape from macrophage phagocytosis inhibited (Supplementary Fig. S7A–C). Herein, our results indicated that CRISPR-dCas9 based methylation editing may be a potential tool to target LNMAS.

Collectively, our results have demonstrated that downregulation of LNMAS, determined by DNA hypermethylation, orchestrates TWIST1-mediated EMT and STC1-dependent immune evasion to promote LN metastasis (Fig. 7J). Thus, our study reveals the essential role of LNMAS and the underlying mechanisms in LN metastasis, providing potential therapeutic targets for CCa patients.

## Materials and methods

### Datasets

The RNA-seq data, clinicopathological data and DNA methylation data of 304 CCa patients were downloaded from TCGA (https://portal.gdc.cancer.gov/). CCa patients were divided into LN metastasis positive group (LNpos, 60 cases in total) and LN metastasis negative group (LNneg, 133 cases in total) according to the TNM staging. The lncRNA expression matrix were obtained based on the gene classification of GENCODE V35 (https://www.gencodegenes.org/). Low-abundance lncRNAs (average cout < 1 and missing values > 10%) were removed and a total of 3778 lncRNAs were included for subsequent analysis.

The lncRNA microarray data were reported before [59]. Briefly, the lncRNA expression matrix of 6 normal cervical tissues (NC) and 5 CCa tissues were obtained by Agilent lncRNA microarray. Seqmap was used to reannotate the lncRNA probes according to the transcriptome annotation files from GENCODE V35.

### Clinical specimens

A total of 26 normal cervical tissues, 56 CCa specimens were recruited for RNA isolation. The samples were frozen immediately in liquid nitrogen and stored at −80 °C. Another 187 paraffin-embedded CCa tissues, collected from January 2006 to December 2012, were obtained from the archives of the pathology department in the First Affiliated Hospital of Sun Yat-sen University. All CCa patients were enrolled with the following inclusion criteria: (1) patients who were staged as IA2 to IIA2 (FIGO 2009) and underwent radical hysterectomy and lymphadenectomy; (2) patients who were pathologically diagnosed as cervical squamous cell carcinoma, cervical adenocarcinoma or adenosquamous carcinoma; (3) patients without radiotherapy or chemotherapy before surgery. Normal cervical tissues were collected from patients who underwent hysterectomy for nonmalignant conditions. Written informed consent was obtained from each patient with approval by the Institutional Review Board of the First Affiliated Hospital of Sun Yat-sen University in accordance with the Declaration of Helsinki.

### Cell culture

CCa cell lines (SiHa and HeLa) were purchased from American Type Culture Collection (ATCC, USA) and cultured following the recommended instructions. HLEC were purchased from ScienCell Research Laboratories and maintained in endothelial cell medium (ScienCell, USA). All cells were cultured in a humidified incubator (Thermofisher, USA) with 5% CO2 at 37 °C. All cell lines were authenticated by STR profiling.

### RNA extraction and real-time quantitative PCR (qPCR)

TRIzol reagent (Takara Bio, China) was used to extract total RNA from clinical samples and cells. Nuclear and cytoplasmic fractions of CCa cells were isolated by a PARIS Kit (Ambion, USA). The concentration and quality of RNA were measured by NanoDrop 2000 (ThermoFisher) and reverse transcribed to cDNA. SYBR Green Premix Pro Taq HS qPCR Kit (Accurate Biotechnology (Hunan)Co., Ltd, China) was utilized to perform qPCR according to the manufacturer’s instructions. The results were analyzed using 2 ^− ΔΔCT^ method. The primers used were listed in Supplementary Table S1.

### In situ hybridization (ISH)

After deparaffinization and rehydration, the sections were treated with 20 μg/ml proteinase K (Qiagen, Germany). A double-(5′ and 3′)-digoxin (DIG)-labeled LNMAS probe with locked nucleic acid (LNA) modification (Qiagen) was added and the sections were hybridized at 55 °C for 1 h. Subsequently, the sections were incubated with an anti-digoxin monoclonal antibody (Roche) for 1 h at room temperature. After staining with NBT-BCIP (Roche, Switzerland) and Nuclear Fast Red nuclear counterstain (Vector laboratories, USA), the sections were observed and analyzed. The intensities of LNMAS staining were scored by 0 (no staining), 1 (light blue), 2 (blue), and 3 (dark blue). The percentage of LNMAS positive cancer cells was designated follows [60]: 0 (no positive), 1 (0–30% positive), 2 (30–60% positive), 3 (over 60%). The ISH scores were calculated by multiplying the scores, ranging from 0 to 9. The results were evaluated by two pathologists independently in a blinded manner. Samples with an ISH score ≤4 were defined as low-LNMAS group and samples with an ISH score > 4 were defined as high-LNMAS group.

### Cell transfection and lentivirus transduction

Full-length of LNMAS was identified by RACE (Takara). pLVX -LNMAS plasmid was constructed using the full length of LNMAS and packaged with lentivirus. Cells were then infected and LNMAS overexpressed cells were selected using 2 mg/ml of puromycin (Sigma, USA) for 7 days. X-tremeGENE HP DNA Transfection Reagent (Roche) was used for plasmids transfection and Lipofectamine RNAiMAX (Invitrogen, USA) was used for siRNAs and ASOs according to the manufacturer’s instructions.

### Western blot

RIPA (Beyotime, China) supplemented with PMSF (Beyotime) was used to extract total proteins. We separated the protein aliquots by SDS-PAGE and the results were visualized using UltraSignal ECL Reagent (Millipore, USA). The antibodies used were listed in Supplementary Table S2.

### Cell migration, invasion and wound healing assays

A 24-well plate Transwell system with chambers (8μm pore size, Corning) was utilized for migration assays. 5 × 104 cells were seeded into the upper chamber and cultured for 24 h. Cells in the lower surface of the chambers were fixed using 4% paraformaldehyde (Sangon Biotech) and stained using 0.1% crystal violet (Beyotime). Matrigel (BD Science, USA) was added for cell invasion assays. For wound healing assays, cells were seeded into 6-well plates and scratched using a 200 μl pipette tip. The wounds were photographed at 0 and 72 h. The percentages of wound closure were analyzed using Image J.

### Animal study

All animal studies were approved by the Institutional Animal Care and Use Committee (IACUC) of Sun Yat-sen University. Animal models were constructed as described before [61, 62]. Briefly, 4-week-old female BALB/c nude mice were purchased from the Experimental Animal Center of Sun Yat-sen University. Mice were fixed and anesthetized by intraperitoneally injection of pentobarbital sodium. For popliteal LN metastasis models, 1 × 10^6^ cells suspended in 0.05 ml PBS were slowly injected into the paw pads. For subcutaneous xenograft tumor models, 5 × 10^6^ cells suspended in 0.5 ml PBS were slowly injected into the dorsal side of the necks. For lung metastasis models, 1 × 10^6^ cells suspended in 0.1 ml PBS were slowly injected into the tail veins. 28 days later, the mice were sacrificed to obtain the primary tumors in paw pads, popliteal LNs, subcutaneous xenografts and lung tissues. Only female mice were used in the CCa mouse models for sex is not the biological variable. Simple randomization was used to allocate 4–8 mice into different groups and no blinding was done.

### RNA sequencing (RNA-seq)

Total RNA was extracted from LNMAS overexpressed SiHa cells and corresponding control cells. The quantity and purity were evaluated with Agilent Bioanalyzer 2100 (Agilent, USA). Poly(A) RNA was purified from total RNA poly-T oligo-attached magnetic beads (New England Biolabs, USA). RNA library construction and high-throughput sequencing were conducted in Genewiz (Suzhou, China). DEGs were identified by Deseq2 and annotated by Metascape.

### Macrophage culture and in vitro phagocytosis assays

3–5 mL venous blood were collected from healthy volunteers. Peripheral blood monocytes were isolated by Ficoll-Hypaque (Tian Jin Hao Yang Biological Manufacture, China) at 400 g density gradient centrifugation for 40 min. 1 × 10^6^ cells resuspended in 0.5 ml RPMI1640 medium were seeded in 24-well plates and stimulated with 100 ng/ml human M-CSF (Sinobiological, China) for macrophage differentiation. Non-adherent cells were removed by repeated gentle washing with warm medium 6 days later.

For in vitro macrophage phagocytosis assays, 1 × 10^5^ macrophages were labeled with 5 μM red fluorescent probe DiI (Beyotime) and seeded in a transparent 24-well plate. 1 × 10^5^ tumor cells were labeled with 5 μM green fluorescent probe CFSE (Topscience, China) and seeded in the same well. Macrophages and tumor cells were cocultured for 4 h. The results were observed under a fluorescent inverted microscope (Leica, Germany) and detected by flow cytometry (Beckman, USA).

### Chromatin isolation by RNA purification (ChIRP)

ChIRP was performed as previously described [63]. Briefly, 2 × 10^7^ cells were crosslinked in 1% formaldehyde at room temperature for 10 min and sheared to 100–500 bp fragments at 4 °C. Biotin-labeled probes were hybridized to target lncRNA and chromatin complexes were purified by magnetic streptavidin beads. Probes targeting Lacz were used for negative control. LNMAS-binding DNAs were further isolated and sequenced. Probes utilized are shown in Supplementary Table S1.

### Chromatin immunoprecipitation (ChIP)

The EZ-Magna ChIP A/G kit (Millipore) was used to perform the ChIP assays according to the manufacturer’s instructions. The immunoprecipitated DNAs were quantified by qPCR. Antibodies utilized are shown in Supplementary Table S2.

### RNA pull-down and mass spectrum

The Magnetic RNA-Protein Pull-Down Kit (Thermofisher) was used to perform the RNA pull-down assays according to the manufacturer’s instructions. The antisense of LNMAS was used as the negative control. The proteins pulled down were detected by protein mass spectrum and verified by western blot.

### RNA immunoprecipitation (RIP) and co-immunoprecipitation (CoIP)

The EZ-Magna RNA-Binding Protein Immunoprecipitation Kit (Millipore) and Protein A/G Plus Agarose Immunoprecipitation Kit (Sangon Biotech) were used to perform the RIP and CoIP assays according to the manufacturer’s instructions. The immunoprecipitated RNAs and proteins were detected by qPCR and western blot, respectively. Antibodies utilized are shown in Supplementary Table S2.

### In vitro methylation assays and dual-luciferase reporter assays

The promoter sequence of LNMAS was cloned into the PGL3 vector (LNMAS-PGL3). LNMAS-PGL3 was methylated in vitro using the CpG Methyltransferase M.SssI (New England Biolabs) by incubation at 37 °C for 12 h, followed by inactivation of enzyme at 60 °C for 20 min. The methylation status was further verified by HpaII digestion and 2% agarose gel electrophoresis.

Cells were transiently transfected with unmethylated or methylated LNMAS-PGL3 plasmids. After 48 h, the luciferase activities were detected using the Dual-Luciferase Reporter Assay System (Promega, USA) according to the manufacturer’s instructions. The luciferase activities were normalized to the renilla luciferase activities. Data were displayed as fold change over the control group.

### CRISPR-dCas9 based DNA methylation editing

CRISPR-dCas9 based DNA methylation editing was conducted as previously reported [32, 34]. Briefly, sgRNAs were designed and inserted into the dCas9-TET1-CD vector (Addgene, USA). Cells were transiently transfected with the constructs and the expression level of LNMAS was detected by qPCR.

### Statistical analysis

R version 3.6 and Graphpad Prism version 7 were used for statistical analysis according to the assumptions of the tests. Student’s *t* test and ANOVA test were used to compare continuous data between two groups and multiple groups, respectively. K-M method and cox regression were utilized to evaluate the cumulative survival rate, and chi-square test was utilized to analyze the correlation between lncRNA expression and clinicopathological parameters. Each experiment was repeated in triplicate. *P* less than 0.05 was considered to be statistically significant.

## Supplementary information

Supplementary Figures

Supplementary Tables
