# Supplementary material for: Downregulation of LNMAS orchestrates partial EMT and immune escape from macrophage phagocytosis to promote lymph node metastasis of cervical cancer
Source: Oncogene. 2022 Feb 12;41(13):1931–43. doi: 10.1038/s41388-022-02202-3 (PMC8956512; doi:10.1038/s41388-022-02202-3)
Supplement: Supplementary file 1 — Supplementary Figures [file 41388_2022_2202_MOESM1_ESM.pdf]

Figure S1

A

Tool	Score	Protein coding potential	Classification
CPC2	0.33	0.033	Noncoding
CPAT	1.21	0.10	Noncoding

B

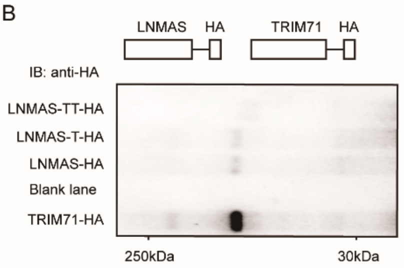

Figure S1. The protein-coding capability of LNMAS. A. The protein-coding capability predicted by CPC2 and CPAT. B. Western blot using anti-HA to detect the protein coded by LNMAS in all three codon phases. TRIM71-HA was used as the positive control.

Figure S2

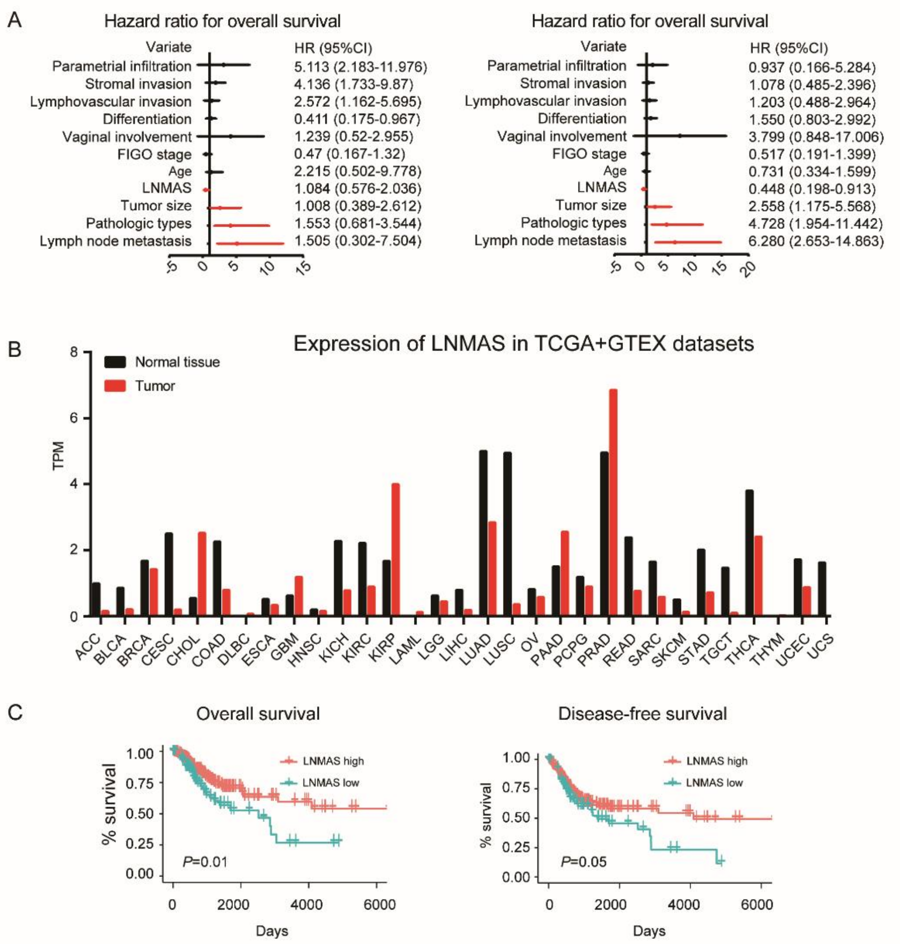

Figure S2. LNMAS predicts a better prognosis in cervical cancer. A. The results of multivariate cox proportional hazards analyses of OS and DFS based on the LNMAS ISH score in our center. B. The expression of LNMAS in human cancers using GEPIA based on TCGA and GTEX datasets. C. K-M analyses of OS and DFS grouped by LNMAS in cervical cancer patients of TCGA dataset.

Figure S3

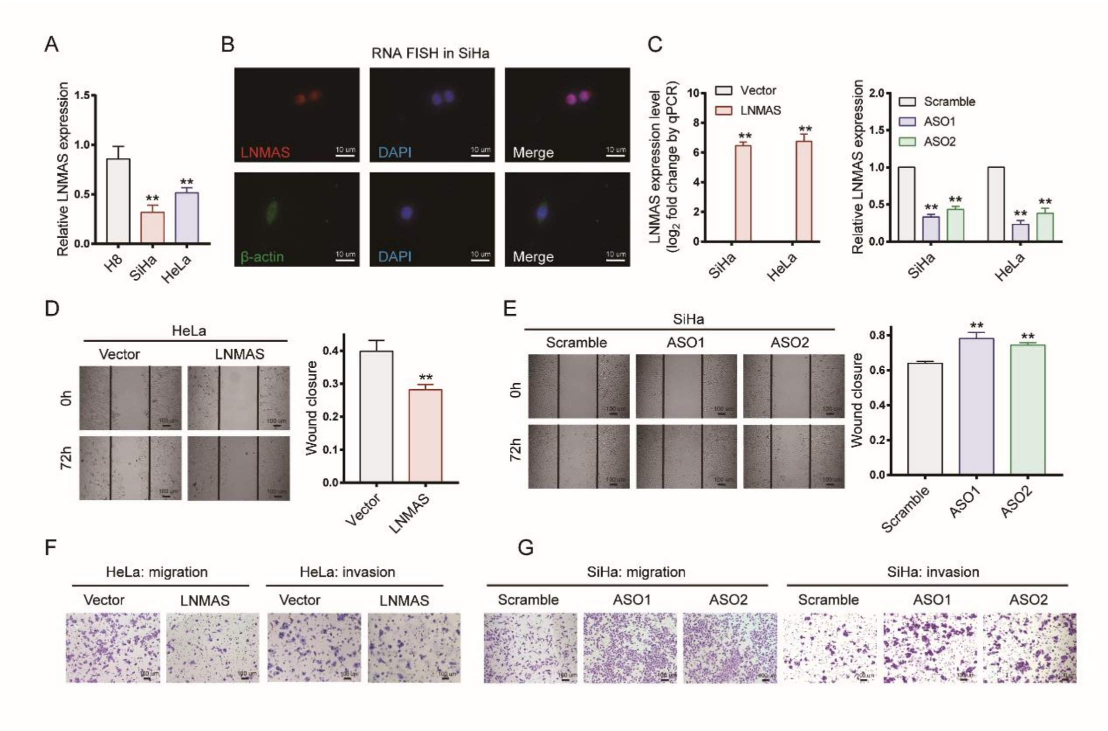

Figure S3. LNMAS inhibits cervical cancer cells metastasis in vitro. A. The relative expression of LNMAS in H8, SiHa and HeLa qualified by qPCR. B. Representative RNA FISH images of LNMAS and β-actin in SiHa. C. The relative expression of LNMAS qualified by qPCR in SiHa and HeLa with LNMAS overexpression or knockdown. D, E. Representative images of wound healing assays and dot plots of wound closure using HeLa and SiHa after overexpression (D) or knockdown (E) of LNMAS. F, G. Representative images of transwell assays using HeLa and SiHa after overexpression (F) or knockdown (G) of LNMAS.

Figure S4

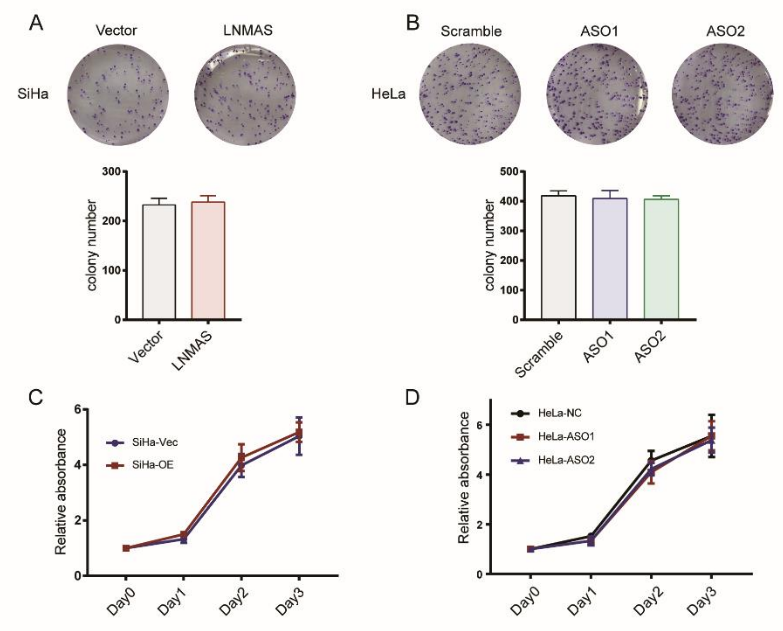

Figure S4. LNMAS has no significant effects on cervical cancer cells proliferation in vitro. A, B. Representative images of colony formation assays using SiHa and HeLa after overexpression (A) or knockdown (B) of LNMAS. C. The results of CCK8 assays using SiHa and HeLa after overexpression (C) or knockdown (D) of LNMAS.

Figure S5

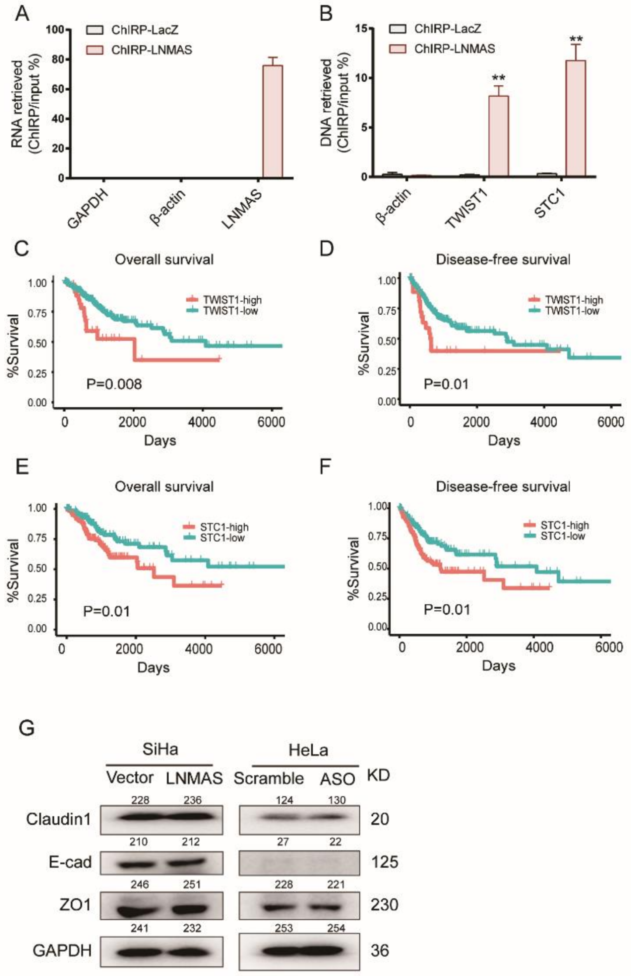

Figure S5. TWIST1 and STC1 are the direct targets of LNMAS. A. QPCR of the retrieved RNA of GAPDH, β-actin and LNMAS by ChIRP in the indicated group. B. QPCR of the retrieved DNA of β-actin, TWIST1 and STC1 by ChIRP in the indicated group. C, D. K-M analyses of OS (C) and DFS (D) grouped by TWIST1 in cervical cancer patients of TCGA dataset. E, F. K-M analyses of OS (E) and DFS (F) grouped by STC1 in cervical cancer patients of TCGA dataset. G. The western blot results of epithelial markers when LNMAS was overexpressed or knocked down.

Figure S6

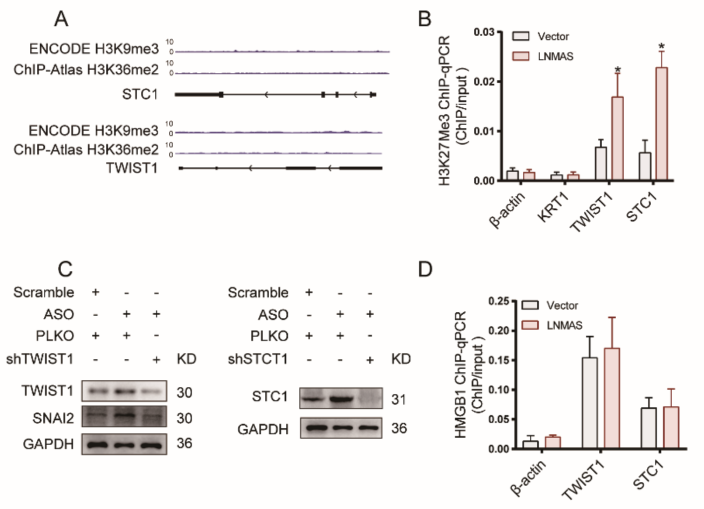

Figure S6. A. The enrichment of H3K9me3, H3K36me2 in the promoters of STC1 and TWIST1 from ENCODE and ChIP-Atlas were shown. B. The ChIP-qPCR results of H3K27me3 in the promoters of STC1 and TWIST1. *: P<0.05. C. The western blot results of TWIST1, SNAI2 and STC1 in the indicated groups. D. The ChIP-qPCR results of HMGB1 in the promoters of STC1 and TWIST1.

Figure S7

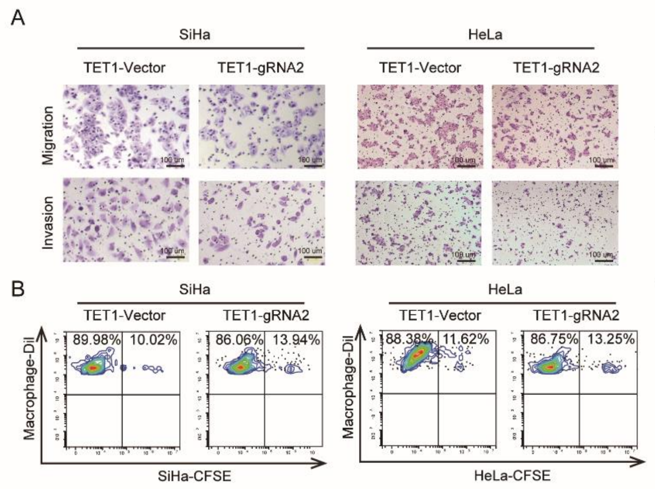

Figure S7. A. The migration and invasion results when cervical cancer cells were demethylated with CRISPR-dcas9. B. The macrophage phagocytosis results when cervical cancer cells were demethylated with CRISPR-dcas9. C. The western blot results of TWIST1, STC1 and other mesenchymal markers in the indicated groups.
