# Supplementary material for: Downregulation of LNMAS orchestrates partial EMT and immune escape from macrophage phagocytosis to promote lymph node metastasis of cervical cancer
Source: Oncogene. 2022 Feb 12;41(13):1931–43. doi: 10.1038/s41388-022-02202-3 (PMC8956512; doi:10.1038/s41388-022-02202-3)
Supplement: Supplementary file 2 — Supplementary Tables [file 41388_2022_2202_MOESM2_ESM.docx]

**Supplementary tables**

Table S1. The sequence of oligos used in the study.

| Name | Sequence (5’-3’) |
| --- | --- |
| LNMAS-F | AGAGGACAGAAGCTTATGGAAAGA |
| LNMAS-R | ATGAACTCCTGAGCCTGCAC |
| HOTAIR-F | GGTGAAAAAGCAACCACGAAGC |
| HOTAIR-R | ACATAAACCTCTGTCTGTGAGTGCC |
| U6-F | CTCGCTTCGGCAGCACA |
| U6-R | AACGCTTCACGAATTTGCGT |
| STC1-F | AAGATGGCGACCACCAAAGT |
| STC1-R | GCAGTGACGCTCATAAGGGA |
| TWIST1-F | TCAAGAGGTCGTGCCAATCA |
| TWIST1-R | TTGCAGGCCAGTTTGATCCC |
| GAPDH-F | ATCACCATCTTCCAGGAGCGA |
| GAPDH-R | CCTTCTCCATGGTGGTGAAGAC |
| SNAI2-F | CGAACTGGACACACATACAGTG |
| SNAI2-R | CTGAGGATCTCTGGTTGTGGT |
| ZEB1-F | ACCCTTGAAAGTGATCCAGC |
| ZEB1-R | CATTCCATTTTCTGTCTTCCGC |
| SNAI1-F | TCGGAAGCCTAACTACAGCGA |
| SNAI1-R | AGATGAGCATTGGCAGCGAG |
| FN1-F | CGGTGGCTGTCAGTCAAAG |
| FN1-R | AAACCTCGGCTTCCTCCATAA |
| MMP1-F | AAAATTACACGCCAGATTTGCC |
| MMP1-R | GGTGTGACATTACTCCAGAGTTG |
| MMP2-F | TACAGGATCATTGGCTACACACC |
| MMP2-R | GGTCACATCGCTCCAGACT |
| MMP9-F | TGTACCGCTATGGTTACACTCG |
| MMP9-R | GGCAGGGACAGTTGCTTCT |
| VEGFC-F | GGCTGGCAACATAACAGAGAA |
| VEGFC-R | CCCCACATCTATACACACCTCC |
| CDH1-F | ATTCTGATTCTGCTGCTCTTG |
| CDH1-R | AGTAGTCATAGTCCTGGTCTT |
| CDH2-F | TCAGGCGTCTGTAGAGGCTT |
| CDH2-R | ATGCACATCCTTCGATAAGACTG |
| CD47-F | TCCGGTGGTATGGATGAGAAA |
| CD47-R | ACCAAGGCCAGTAGCATTCTT |
| SLAMF7-F | ACAACCCCTCTTGTCACCATA |
| SLAMF7-R | CCCACATAGTAGATCCCTGAGTC |
| CD274-F | TGGCATTTGCTGAACGCATTT |
| CD274-R | TGCAGCCAGGTCTAATTGTTTT |
| CALR-F | CCTGCCGTCTACTTCAAGGAG |
| CALR-R | GAACTTGCCGGAACTGAGAAC |
| CD24-F | CTCCTACCCACGCAGATTTATTC |
| CD24-R | AGAGTGAGACCACGAAGAGAC |
| B2M-F | GAGGCTATCCAGCGTACTCCA |
| B2M-R | CGGCAGGCATACTCATCTTTT |
| LNMAS-ChIRP-probe1 | CTGCTCACTGCTGACTGCTT |
| LNMAS-ChIRP-probe2 | CAATCTTGCAGGGTTAGTTC |
| LNMAS-ChIRP-probe3 | ATAAGCTTCTGTCCTCTTTG |
| LNMAS-ChIRP-probe4 | TGGAGTTGGTGTGGCTCCTG |
| LNMAS-ChIRP-probe5 | CACACAGCAAATCTATGCCA |
| LNMAS-ChIRP-probe6 | TGAGGCCCAGCAAGGGAAAG |
| LNMAS-ISH-probe | TCAGGTCTTGGTTAATGATCA |

Table S2. The antibodies used in the study.

| Antibody | Catalog# | Working concentration | Manufacturer |
| --- | --- | --- | --- |
| TWIST1 | 25465-1-AP | 1:1000 | Proteintech |
| SNAI2 | 12129-1-AP | 1:1000 | Proteintech |
| ZEB1 | 21544-1-AP | 1:1000 | Proteintech |
| FN1 | 15613-1-AP | 1:1000 | Proteintech |
| MMP2 | 10373-2-AP | 1:1000 | Proteintech |
| VEGFC | sc-374628 | 1:1000 | Santacruz |
| GAPDH | AP0063 | 1:5000 | Bioworld |
| CD47 | sc-12730 | 1:1000 | Santacruz |
| LYVE-1 | ab14917 | 1:50 | Abcam |
| Pan-Keratin | 4545S | 1:500 | Cell Signaling Technology |
| HMGB1 | 10829-1-AP | 1:1000 | Proteintech |
| H3K27ac | 39133 | 1:100 | Active Motif |
| H3K4me3 | 39159 | 1:100 | Active Motif |
| BRG1 | 21634-1-AP | 1:1000 | Proteintech |

Table S3. The promoter regions of TWIST1 and STC1 for promoter reporter constructs

>TWIST1: GRCh38: chr7: 19115820:19117820: -1

GGGAGGACGAATTGTTAGACCCCGAGGAAGGGAGGTGGGACGGGGGAGGGGGACTGGAAAGCGGAAACTTTCCTATAAAACTTCGAAAAGTCCCTCCTCCTCACGTCAGGCCAATGACACTGCTGCCCCCAAACTTTCCGCCTGCACGGAGGTATAAGAGCCTCCAAGTCTGCAGCTCTCGCCCAACTCCCAGACACCTCGCGGGCTCTGCAGCACCGGCACCGTTTCCAGGAGGCCTGGCGGGGTGTGCGTCCAGCCGTTGGGCGCTTTCTTTTTGGACCTCGGGGCCATCCACACCGTCCCCTCCCCCTCCCGCCTCCCTCCCCGCCTCCCCCGCGCGCCCTCCCCGCGGAGGTCCCTCCCGTCCGTCCTCCTGCTCTCTCCTCCGCGGGCCGCATCGCCCGGGCCGGCGCCGCGCGCGGGGGAAGCTGGCGGGCTGAGGCGCCCCGCTCTTCTCCTCTGCCCCGGGCCCGCGAGGCCACGCGTCGCCGCTCGAGAGATGATGCAGGACGTGTCCAGCTCGCCAGTCTCGCCGGCCGACGACAGCCTGAGCAACAGCGAGGAAGAGCCAGACCGGCAGCAGCCGCCGAGCGGCAAGCGCGGGGGACGCAAGCGGCGCAGCAGCAGGCGCAGCGCGGGCGGCGGCGCGGGGCCCGGCGGAGCCGCGGGTGGGGGCGTCGGAGGCGGCGACGAGCCGGGCAGCCCGGCCCAGGGCAAGCGCGGCAAGAAGTCTGCGGGCTGTGGCGGCGGCGGCGGCGCGGGCGGCGGCGGCGGCAGCAGCAGCGGCGGCGGGAGTCCGCAGTCTTACGAGGAGCTGCAGACGCAGCGGGTCATGGCCAACGTGCGGGAGCGCCAGCGCACCCAGTCGCTGAACGAGGCGTTCGCCGCGCTGCGGAAGATCATCCCCACGCTGCCCTCGGACAAGCTGAGCAAGATTCAGACCCTCAAGCTGGCGGCCAGGTACATCGACTTCCTCTACCAGGTCCTCCAGAGCGACGAGCTGGACTCCAAGATGGCAAGCTGCAGCTATGTGGCTCACGAGCGGCTCAGCTACGCCTTCTCGGTCTGGAGGATGGAGGGGGCCTGGTCCATGTCCGCGTCCCACTAGCAGGCGGAGCCCCCCACCCCCTCAGCAGGGCCGGAGACCTAGGTAAGGACCGCGCCGCTGCACCCCTTCGCCTCTCAGGTGGCAGACGGCAGGCCGGCCAGGCCGCGGTTCCCAGTCCACCTCGATTTCCTCCCCTCTCCCACTCTCCGCTCAGCCTTCCCACCTCACTTGGCACCGTTGCCTCGCGCCCCCAGCGTCCCCGGAAGGCCGGTCTGACCCCGCTAGGGAGAGCAGTCTCCAGGGGGATGCGCCCTGGTGAGGGGTGTGTGTGCGCGTGAGTGTGCGTGACAGGAGGGGAGACAGAGACACCCAGGGTCACGGGTAAGGACCGTTTTGTCAGCGCCACCCTTTCTTTCGGCTTTCAATTTTTGTTCTCCTTAAAACAAATGTTTTAAAACAAATTCCACCTCCTCCTCCTTTCCACCCACCCACTTCCTCTTGCCCTTGGGCTGAAATCCTTCCAGGTTGTTCAGCTTAATTTCTCAGTGGTGGTGATAAGAACAGTGCTCACTAGTCTTAGAAAACAGCCGCAGAGACCTAAACAATAACCGACTCCCCCCCCCCCCTCTGGGTTTTTGCAGATGTCATTGTTTCCAGAGAAGGAGAAAATGGACAGTCTAGAGACTCTGGAGCTGGATAACTAAAAATAAAAATATATGCCAAAGATTTTCTTGGAAATTAGAAGAGCAAAATCCAAATTCAAAGAAACAGGGCGTGGGGCGCACTTTTAAAAGAGAAAGCGAGACAGGCCCGTGGACAGTGATTCCCAGACGGGCAGCGGCACCATCCTCACACCTCTGCATTCTGATAGAAGTCTGAACAGTTGTTTGTGTTTTTTTTTTTTTTTTTTTTGACGAAGAATGTTTTTATTTTTATTTTTTTCATGCATGCAT

>STC1: GRCh38: chr8: 23853193:23855193: -1

TAAAGAGATCACATTTCCCCACCATACCCCTGCTATCCATTTCCCCCAAGTGGCTCATTAGAAAAAAAGATGGCTAGATTTCAAAAAGCAACTTGGAGAGATTTCTATAGGATTTTTCTTTAGTTCAATCAATACAGAGTTATCTCTTACTTCCACGAAAATAGCTTTTTCACACATCTCTGCACACACAGTCACACACACATATAAAACATTGGCAGCAGGTACTTTTAATTTGCTGGAAAATATTTCTAAGAAGTCAAAAAGCTCCAGCTGAATTGCATGCCCTCTTATTGGCTCACCAGACCAGTTGAGGGACCTGATTGGTCCTTGATCCTGAGGACCGATAAGAACGGCTATAAAATCCCTGGGTGCAGCTCTTGGGCCCCCAGTTTGCAAAAGCCAGAGGTGCAAGAAGCAGCGACTGCAGCAGCAGCAGCAGCAGCGGCGGTGGCAGCAGCAGCAGCAGCGGCGGCAGCAGCAGCAGCAGCGGAGGCACCGGTGGCAGCAGCAGCATCACCAGCAACAACAACAAAAAAAAATCCTCATCAAATCCTCACCTAAGCTTTCAGTGTATCCAGATCCACATCTTCACTCAAGCCAGGAGAGGGAAAGAGGAAAGGGGGGCAGGAAAAAAAAAAAACCCAACAACTTAGCGGAAACTTCTCAGAGAATGCTCCAAAACTCAGCAGTGCTTCTGGTGCTGGTGATCAGTGCTTCTGCAACCCATGAGGCGGAGCAGAATGACTCTGTGAGCCCCAGGAAATCCCGAGTGGCGGCTCAAAACTCAGGTAAGCAGCAAACCCAAGAGCGGTTTCTCCCCCAAAGAGCTGTCCTCATTTGCCTCTCCCTTTTGCAACTGTGTCTGTGACGGCTGATCTTGATAATAAATGTGCTTCATGCCTGATGGCAATAAACTGCCAGTGTAATCCAATAGCCTTAGGCAGTGGAGCTCCTTTGTTTAATAAATTGCATGCAACTAAACGAAGAAGCTGGACGCTCTGCTGAGGGTATTTCATTGCATAAGCCTAGCCTGATTGCCTGAAATCTGGCACGTACCCTCTTGGAGGGGGAGGGGTGAGAGGGGAGGAAAGGCTTGAATGTTGGCATGCTTCAAAGCGCTCTCTATACTTTCCAGAAGCTGATCTAAGGTAAGACCTGGCTTGTTTGATGCTGTCCCTCTTCCTTTCCTCTTAAGACTCTACCTCTTCACTTCTTGACTTCTCTACCTAAGAGAATACACAGAAAAGCTCCCTCTTCAGACTGATTTTCAGAACCATAGCCCTCAAGTCTGACAAATCCAGGGCGGATTCAGAGAAAACCCTATACATCCCACCCCCAACCCTGCTGCCTGTCTCCCTCCTGGCTCTGTAGGGCTCCATTCAGTCGTCCCTGCAGCCCGTAGCCAGAGAGCCCCTAGAAAAAAAAAATTGTGAAACGTAGCAGCTGTTTCTCCAAGGGTAAGGTCTCACTTTCGTCTTGACTCTTGGAGCAGTTAATTGTGCATTATCTTGGCTTCTGAAGGAAAAGAATCATTAGGAAGCCTGCATTGACACCTCTGCCTCTTGACTTCTCTGGCTCCACTTGTTCCCCTTCCAATACTGCCCTCTGTCCTCCAACTGTCAGCAAAGACCGGATCACAGGTGTCAAAATGCTGCAGATGATGATCCCCTTGGAAGAGCACAGGTCCCTGCCGAGAGGAGGGCTTTTGCTTCACTTCCTTTTGTACTTAGTTGCTATAGAGATGCAGTGATTCACAATTCGAGCTTGCCTAAAACAATAGCAGCCCTAATGCATATTAAAACTGCTTAAAGCAAAGTTATAATTTAAACCCGTGGAAATATATAATTAGGGAAATGACTTGTAACATCCTAGAGGCTGCGGGTGGTGGGAGGGGGTGGGGAAGACTAACTGAACCATGCTTTTGATCTCAGAGGAGAATCCTATGGCATGAAGGCTGCAGATCATTCCTTGCCTAGCTGCCTGCCAGAACACCCCCCTTCT
